# The untapped potential of guideline-directed heart failure pharmacotherapy: consequences of missed opportunities

**DOI:** 10.1093/ehjcvp/pvae096

**Published:** 2024-12-24

**Authors:** Ester J Herrmann, Samuel Sossalla

**Affiliations:** Medical Clinic I, Cardiology and Angiology, Justus-Liebig-University, Klinikstrasse 33, 35392 Giessen, Germany; Medical Clinic I, Cardiology and Angiology, Justus-Liebig-University, Klinikstrasse 33, 35392 Giessen, Germany; Department of Cardiology, Kerckhoff-Clinic, Benekestrasse 2-8, 61231 Bad Nauheim, Germany; German Center for Cardiovascular Research (DZHK), Partner Site Rhine-Main, Partner Site Rhine-Main, Benekestrasse 2-8, 61231 Bad Nauheim, Germany

The european society of cardiology (ESC) guidelines for the diagnosis and treatment of heart failure (HF) stipulate that all patients, particularly those experiencing acute decompensation of HF and recent hospitalization, should undergo initiation and rapid up-titration of the four essential HF medications, whenever feasible. Although this approach is crucial for mitigating the risk of HF hospitalization and death, real-world clinical practices often fall short, resulting in missed opportunities that must be urgently addressed.

A recent hospitalization for HF increases the likelihood of initiating guideline-directed medical therapy (GDMT) by 200–400%. However, the benefits of this initiation are often short-lived, with discontinuation rates between 40% and 60%, and up to 44% of patients receiving no GDMT at all.^1^ This reflects significant gaps in care, even with strong evidence supporting the benefits of GDMT.

Utilizing a combination of multiple pharmacological agents and up-titration is associated with a marked reduction in mortality compared with monotherapy.^2^ However, even among advanced HF patients, nearly 50% do not reach target doses of GDMT, despite the absence of contraindications.^3^

The STRONG-HF Trial provides compelling evidence for the benefit of rapid GDMT up-titration. Patients hospitalized for acute HF were randomly assigned for usual care or early and rapid up-titration of HF medications before and within 2 weeks post-discharge. Most importantly, up-titration was only deferred when systolic blood pressure was <95 mmHg, potassium >5.0 mmol/L, or estimated glomerular filtration rate (eGFR) <30 mL/min/1.73 m². The study was halted early for clear benefit, thereby underscoring that this strict concept can effectively improve prognosis.^4^

Despite this overwhelming evidence, several barriers hinder the effective use of GDMT. The lack of structured follow-up programs, limited resources, and timely outpatient appointments often results in missed opportunities for medication adjustment. Furthermore, patient education regarding the importance of GDMT is often insufficient, leading to poor adherence. Renal function and age also affect therapy initiation. Patients with an eGFR of 30–59 mL/min are less likely to receive appropriate GDMT, even without contraindications.

Strategies to improve GDMT adherence and titration may be a tailored drug-layering approach in discharge letters, combined with the involvement of specialized HF nurses. Collaboration with general practitioners, pharmacists, and the use of digital health tools can also reduce clinical inertia and improve care, particularly in outpatient settings.^5^

The evidence for the benefits of early and sustained GDMT is clear, yet clinical uptake remains suboptimal. Addressing this important and prognostic gap will require a comprehensive, patient-centred approach that includes better follow-up, enhanced education, interdisciplinary collaboration, and merits urgent research.

The true tragedy lies in the fact that we already have effective therapies, yet they remain underutilized. The time to act is now—before forging ahead, we must urgently bridge this critical gap in care.

## Data availability

No new data were generated or analysed in support of this research.
